# Prenatal diagnosis of a fetal scalp glomuvenous malformation– a case report

**DOI:** 10.1515/crpm-2024-0014

**Published:** 2026-09-16

**Authors:** Tara Giacchino, Matthew Brown, Bassel Zebian, Istvan Bodi, Alexia Egloff Collado, Ranjit Akolekar

**Affiliations:** Medway Fetal and Maternal Medicine Centre, Medway NHS Foundation Trust, Gillingham, UK; Institute of Medical Sciences, Canterbury Christ Church University, Rowan William’s Court, Kent, UK; Department of Neurosurgery, King’s College Hospital NHS Foundation Trust, London, UK; Department of Clinical Neuropathology, King’s College Hospital NHS Foundation Trust, London, UK; Perinatal Imaging, Centre for Developing Brain, Guy’s and St Thomas’s Hospital, London, UK

**Keywords:** prenatal diagnosis, fetal scalp tumor, glomuvenous malformation

## Abstract

**Objectives:**

Congenital glomuvenous malformations are uncommon vascular maformations caused by mutations in the glomulin gene that are either sporadic or dominantly inherited.

**Case presentation:**

We describe the case of a fetal scalp mass – a glomuvenous malformation – detected prenatally in the third trimester of pregnancy. This defect was an isolated incidental finding without any other intra- or extracranial abnormalities. The pregnancy was managed in a tertiary fetal medicine unit, detailed investigations were performed and a plan for delivery was made following multidisciplinary input. The neonate was delivered following a planned caesarean section at 37–38 weeks. The mass was resected by the neurosurgical team on the first day of life and the baby was discharged in good health on day 7. The mass was sent for neuropathological examination, which confirmed a diagnosis of glomuvenous malformation.

**Conclusions:**

Our case highlights the importance of third trimester assessment of fetal anatomy and the importance of prenatal diagnosis and multidisciplinary teamwork to improve perinatal outcomes in high-risk pregnancies. It also highlights that glomuvenous malformation should be considered in the differential diagnosis in pregnancies presenting with a subcutaneous scalp mass without an accompanying calvarial defect.

## Introduction

Fetal masses related to head and neck are uncommon [1]. Extracranial masses in the fetal head are usually associated with a calvarial defect in the midline and present clinically as an encephalocele. These could be a meningocele - a herniation of meninges containing cerebrospinal fluid or a myelomeningocele - a herniation of brain tissue and cerebrospinal fluid. Extracranial fetal head masses without a calvarial defect are infrequent and include a differential diagnosis of lipomas, epidermoid/dermoid cysts, hamartomas and tumors such as teratomas [2], [3], [4]. Fetal tumors, whether benign or malignant, are uncommon and the reported incidence ranges from about 2-13 per 100,000 livebirths [5]. Tumors in the fetal head are even less common. A systematic review by Tonni et al., looked at 306 cases of prenatal diagnosed fetal tumours of the head and neck and found that the gestational age at diagnosis was mostly in the 3rd trimester of pregnancy between 27 and 34 weeks of gestation. They found the tumours most frequently detected in the head and neck were haemangiomas or lymphangiomas, teratomas, tumours of the gingiva and lymphatic venous malformations [6], 7].

We present a case of a congenital glomuvenous malformation, which was detected as a fetal scalp mass in the third trimester of pregnancy. Glomuvenous malformations are uncommon vascular malformations that are sporadic or dominantly inherited with incomplete penetrance and variable expression secondary to mutations in the glomulin gene. These malformations are secondary to abnormal differentiation of vascular smooth muscle cells, known as glomus cells. The majority of these tumors or masses are detected in the neonatal period after birth and fetal presentations are infrequent, with paucity of studies reporting prenatal diagnosis of such cases [8].

Our case highlights that it is important to keep such infrequent presentations in mind as the outcome of such fetuses and neonates depends on management by specialist teams including Fetal Medicine, Radiology, Obstetric, Neonatology, Neurosurgery and Neuropathology specialists to ensure appropriate and timely treatment.

## Case presentation

A 26-year-old Caucasian primagravid woman was referred to our tertiary Fetal Medicine unit at Medway Fetal and Maternal Medicine Centre with suspicion of a fetal skull mass noted at a transabdominal ultrasound scan performed locally at 29 weeks and 6 days. The pregnancy was following a spontaneous conception. She did not have any significant medical, surgical or family history of note. Her first trimester combined screening for common chromosomal aneuploidies was deemed to be low-risk for trisomy 13, 18 and 21 (<1:20,000 for all three aneuploidies). The anomaly scan at 22 weeks and 4 days demonstrated a normal fetal growth with a normal fetal anatomy and structure. According to standard guidelines for pregnancy scans in the United Kingdom, she was discharged to community care and not booked to have any further scans unless there was a clinical concern. She had a third trimester ultrasound scan at a private clinic for reassurance when it was noted that there was a 43.3 × 38.9 × 48 mm^3^ echogenic mass overlying the fetal skull. Following review of this private scan in her local hospital, a tertiary fetal medicine referral was sent to our unit for further assessment.

**Figure 1:**
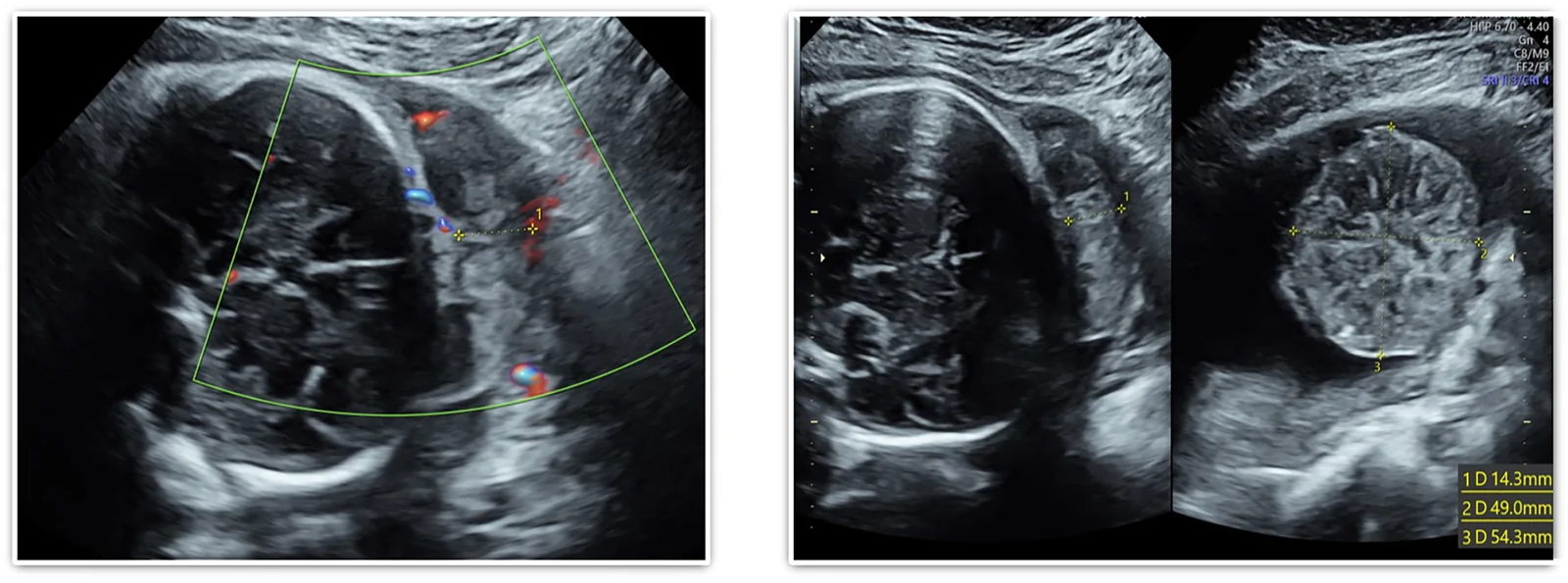
Ultrasound images of the glomovenous malformation (GVM) presenting as a mixed heterogeneous avascular mass in the fetal scalp located on top of the fetal head within the subcuticular tissue and separate to the bone.

**Figure 2:**
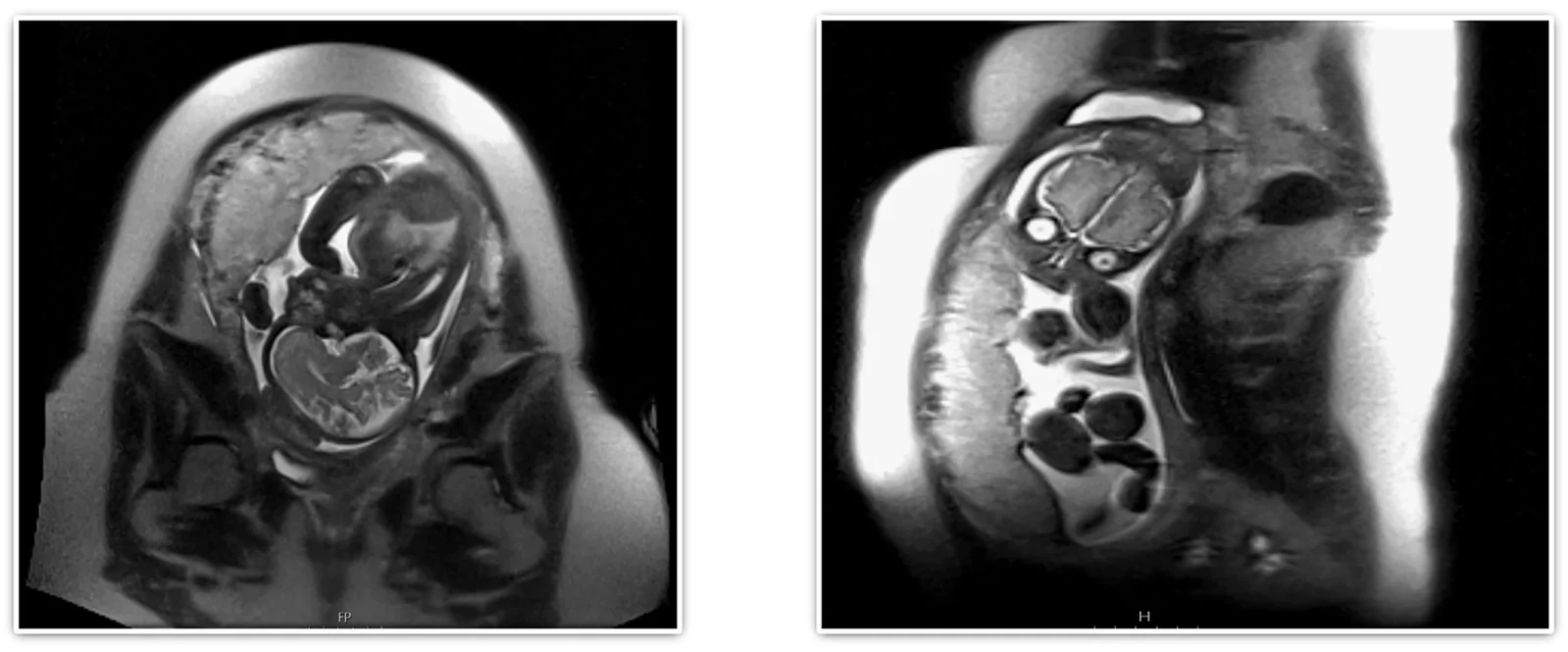
Magnetic resonance images of glomovenous malformation (GVM) on sagittal and coronal views of T2 weighted images demonstrating the scalp soft tissue mass with no cystic changes, no obvious blood vessels and no underlying skull defect.

We reviewed her at 32 weeks and 4 days when we confirmed the findings of normal growth, amniotic fluid volume and Doppler assessments; the ultrasound scan also demonstrated that there was a 51 × 47 × 44 mm^3^ mixed, heterogenous, mass with no Doppler flow in the fetal scalp located in the region of the anterior fontanelle (Figure 1). The rest of the fetal anatomy assessment was normal and the fetal scalp mass was an isolated finding. Detailed fetal neuroanatomical review based on the transabdominal and transvaginal ultrasound scan demonstrated a normal intracranial anatomy and no calvarial defect. The facial profile was normal. The scalp mass appeared to be located subcutaneously over the parietal bones spread equally across the saggital suture on either side. Doppler assessment of the mass did not demonstrate any flow, neither within the mass nor in its periphery and there was no evidence of any extension of the dural venous sinus into the mass, which was contained within the fetal cranial cavity. A diagnosis was made of a fetal subcutaneous scalp mass with a differential diagnosis of hematoma, lipoma, haemangio-lymphoma or Sturge Webber syndrome. The fetal alloimmune thrombocytopaenia (FAIT) screen for human platelet antigens reported that there was no evidence of any alloimmune platelet incompatibility between the parents, thus ruling out fetal thrombocytopaenia as an underlying cause. The fetal MRI scan demonstrated similar findings to the ultrasound scan confirming an intact calvarium and presence of a subcutaneous mass measuring 55 × 48 × 13 mm^3^ over the fronto-parietal region, with no evidence of any cystic changes nor presence of any fetal blood vessels within the mass. The MRI also confirmed that the intracranial venous system was normal with no extension of the dural venous sinus into the mass (Figure 2). The rest of the fetal brain anatomy, including myelination and sulcation appeared normal for gestational age. The neuroradiology differential diagnosis was that of a haemangioma or a teratoma. The pregnancy was closely monitored with ultrasound scans in our fetal medicine unit to assess for any changes in the size, appearance, or vascularity of the scalp mass. The mass findings remained relatively stable over the course of the pregnancy. A fetal neurosurgical review was sought and the recommendation was to continue antenatal monitoring, delivery by Caesarean section at a level 3 neonatal intensive care unit at 37–38 weeks, review by neonatologist following delivery and postnatal neurosurgical consultation to make further management plans regarding need for surgical intervention. Vitamin K administration was recommended orally.

**Figure 3:**
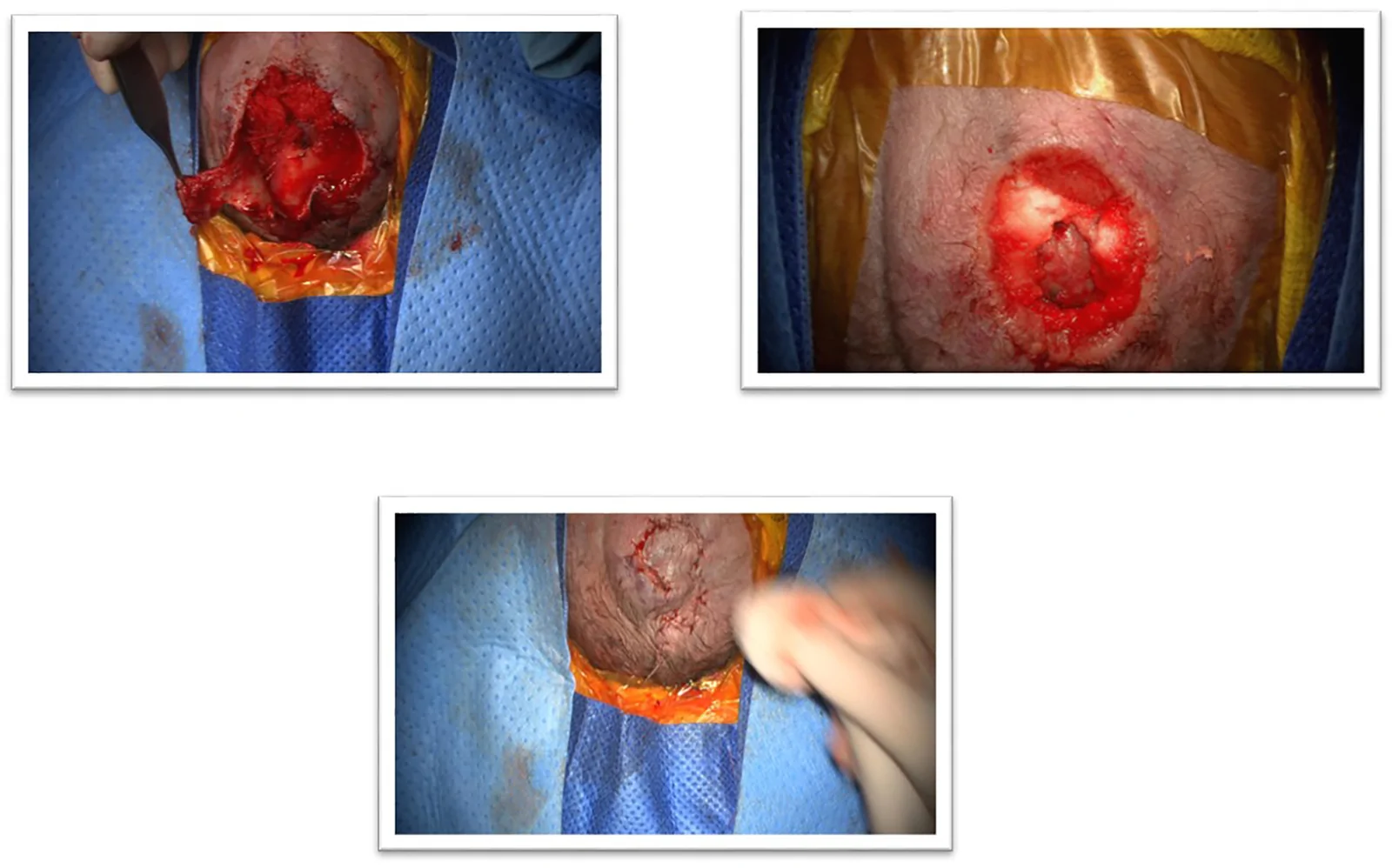
Intraoperative images of the repair of the neonatal scalp defect with bilobed flap.

**Figure 4:**
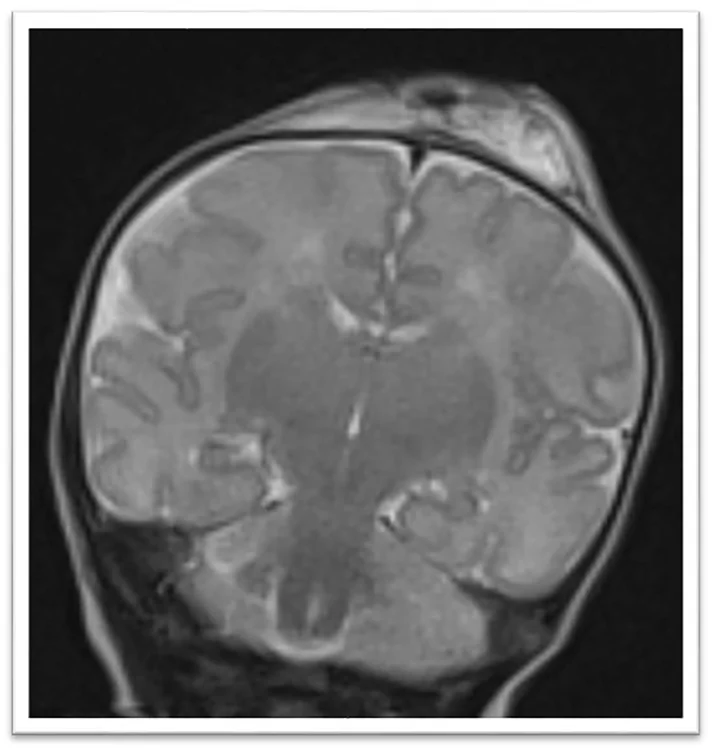
Postnatal coronal T2 image after detachment at birth shows residual heterogeneous soft tissue mass near the vertex with some flow voids and a small amount of haemorrhage. An intact calvarium was seen and intracranial structures, including venous sinuses were normal.

An elective caesarean section was carried out at 37 weeks and 3 days with delivery of a male infant weighing 2,600 g. The Caesarean was uncomplicated and the fetus was delivered easily *en caul* to minimize manipulation of the fetal head during delivery. Despite attempts to mitigate manual handing and trauma, the scalp mass detached almost in its entirety. The baby was born in good condition with Apgar scores of 10, 10 and 10 at 1, 5 and 10 min of life respectively, and the estimated maternal blood loss during Caesarean section was 300 mL. Neonatal examination in the intensive care unit revealed that the defect was oozing and therefore pressure was applied for haemostasis to minimize this. Following initial stabilisation in the neonatal unit, an urgent neonatal transfer was arranged to the tertiary neurosurgical centre for further management. The neonate underwent surgical repair on day 1 of life and was operated on by the paediatric neurosurgeon and a plastic surgeon. The open wound was cleaned and debrided and venous oozing controlled using a haemostatic matrix. The remaining part of the scalp mass was sent for neuropathology examination. A bilobed flap was created and rotated to cover the defect and the skin was closed using absorbable vicryl rapide 4/0 sutures (Figure 3). The neonate received 1 unit of red blood cell transfusion and was monitored over the next few days and no further bleeding or deterioration was noted. The baby was assessed on day 6 post-operatively and clinical examination demonstrated healthy skin flaps and wound edges. The baby was considered fit for discharge on day 7 and outpatient follow up was arranged. The postnatal MRI scan demonstrated post-surgical changes with intact calvarium and intracranial structures (Figure 4).

**Figure 5:**
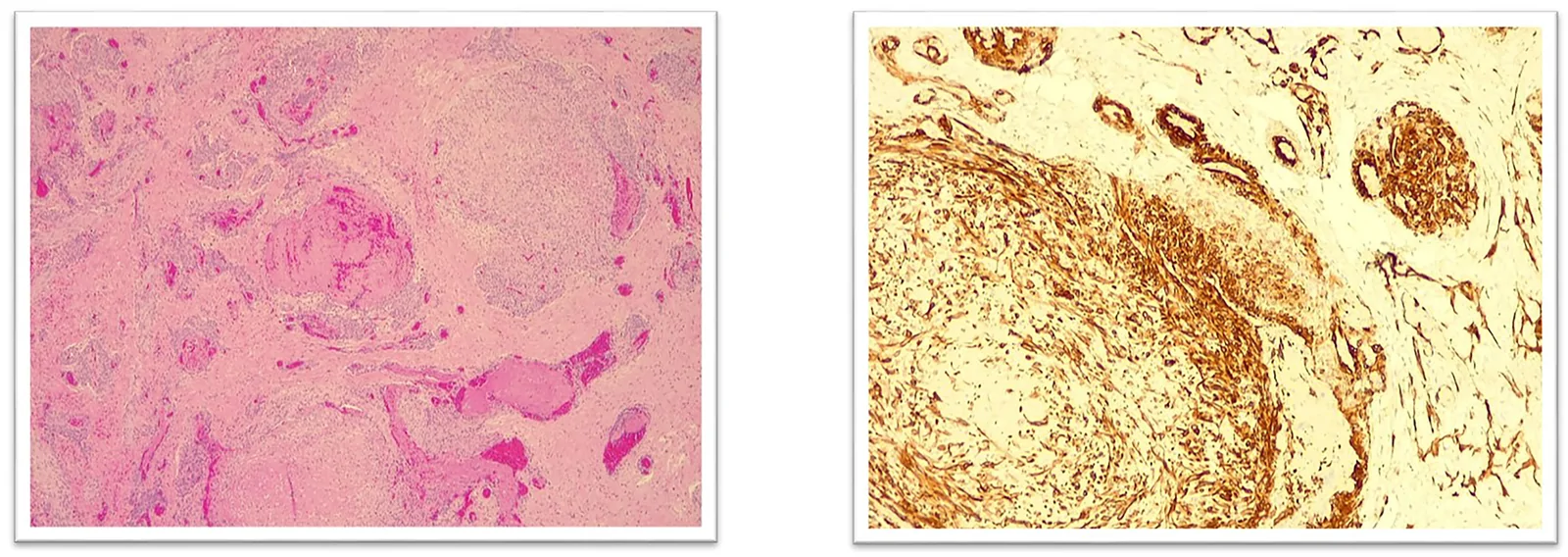
Histological appearances of the glomovenous malformation (GVM) with hematoxylin and eosin (HE) stain and smooth muscle actin-1 (SMA-1) stain.

The preliminary neuropathology report stated that the scalp mass was a benign vasoforming lesion, in keeping with a glomangioma overlapping with glomangiomyoma. However, after detailed neuropathological review, a diagnosis of a Glomuvenous malformation (GVM) was made. Histologically, the GVM’s are made of venous-like channels surrounded by poorly differentiated smooth muscle–like glomus cells that stain positively for smooth muscle actin, similar to glomangiomas (Figure 5). It was noted that the location was unusual and presentation *in utero* uncommon. Immunohistochemistry testing confirmed cells that were CD34 and smooth muscle actin positive but negative for S100, Desmin, Chromogranin, Synaptophysin, EMA, GFAP Stat6, consistent with a diagnosis of a GVM.

## Discussion

Congenital GVM are uncommon vascular maformations caused by mutations in the glomulin gene that are either sporadic or dominantly inherited. Not all cutaneous vessels are affected which supports the idea that the development of lesions likely requires a subsequent event, thought to be a somatic second-hit mutation. The fetal presentation of GVM is even less common. Boon et al., examined a case series of such venous anomalies, 135 of which were GVM (105 inherited and 30 sporadic) and noted that all these lesions presented postnatally after birth or much later in childhood, around the time of puberty [9]. Presentation of a GVM on the scalp is rare as they are usually found on the extremeties, trunk, perinuem or face and neck region, and are typically found at delivery on initial neonatal examination or later in childhood with hardly any reports that describe a prenatal diagnosis [9].

A small case series reported 2 cases of congenital thoracic plaque-type GVM diagnosed in neonatal life, both of whom had a prenatal finding of isolated pleural effusion and ascites. The authors hypothesized a non-random assoication of GVM with pleural effusion and ascites given the common emryologic origin of the superficial cutaneous lesions and deep pleural lining and found that the more severe the skin involvement, the more severe the fetal effusion [10]. Similarly, Tejedor et al., reported on a case of a newborn found to have a congenital plaque like GVM at birth on the inner thigh and legs, with the only prenatal finding being isolated fetal ascites [11]. GVM do not usually involve visceral organs, however, of note are 2 case reports that found a ventricular septal defect in a child with a thoracic plaque like GVM [12] and transposition of the great vessels was found in a boy with multiple nodular GVM [13].

GVM’s have been variably described in literature as glomangiomas, multiple glomus tumor and venous malformation with glomus cells. However, since they are non-neoplastic and are a subtype of venous malformation, the term GVM was proposed in 2002 to describe these malformations [8]. GVMs are classified into solitary or multiple types with a solitary nodule being the most common presentation. The less common multiple lesion type can be further classified into localised, disseminated and congenital plaque form [14], 15]. The exact prevelance of GVMs is unknown as most patients have small asymptomatic lesions. They equally present in both males and females and around 40 % are heritable [8], 9], 16]. GVM are usually found on the extremeties and present as erythematous dark blue nodules and can be painful or painless [17]. Management of GVMs is dependant on the number, site and the amount of progression or dissemination of the lesions. Those that are small and few can be surgically removed, which is curative if the excision is complete [18].

This case highlights a few important issues: First, third trimester ultrasound scans are not only important to assess fetal well-being and planning for delivery by assessing fetal growth, presentation, amniotic fluid volume and Dopplers but they are also important for examination of fetal anatomy as there are some fetal abnormalities or conditions that only present in the third trimester of pregnancy [19], 20]. A large prospective study looked at 104,151 women with a singleton pregnancy to investigate the incidence of fetal anomalies identified at a routine third trimester (35–36 week) ultrasound scan. They found that the incidence of fetal abnormalites detected for the first time during this scan was 0.77 % (803/104,151) which further empahsies that a significant proportion of abnormalities are detected later in pregnancy [21]. It is currently not part of standardised routine practice in the UK to offer third trimester scans between 35 and 37 weeks which leaves a portion of abnormalities undiagnosed prenatally. This could potentially have a significant impact on the perintal outcome, which can be mitigated by effective prenatal diagnosis at the 3rd trimester scan.

The defects identified antenatally in the third trimester require input from a variety of specialists and a timely discussion to plan delivery and postnatal care in order to ensure the best perinatal outcomes in such high-risk pregnancies.

## Conclusions

Our case highlights the importance of a third trimester ultrasound assessment to examine fetal growth and well-being but also to undertake a review of fetal anatomy as there are certain conditions such as microcephaly, gastrointestinal atresias, urogenital defects due to obstruction, abdominal/ovarian cysts and as in our case fetal scalp malformations that will only present in the third trimester. Although uncommon, fetal GVMs should be borne in mind when fetal scalp masses are seen on a third trimester ultrasound, especially in the absence of a fetal calvarial defect. Fetal imaging using ultrasound and MRI is valuable and multidisciplinary consultations involving the fetal/paediatric neurosurgical teams is essential to improve neonatal and paediatric outcomes.
